# Structural Variation of the X Chromosome Heterochromatin in the *Anopheles gambiae* Complex

**DOI:** 10.3390/genes11030327

**Published:** 2020-03-19

**Authors:** Atashi Sharma, Nicholas A. Kinney, Vladimir A. Timoshevskiy, Maria V. Sharakhova, Igor V. Sharakhov

**Affiliations:** 1 Department of Entomology, Virginia Polytechnic and State University, Blacksburg, VA 24061, USA; atashi04@vt.edu (A.S.); v.a.timoshevsky@gmail.com (V.A.T.); msharakh@vt.edu (M.V.S.); 2 Genomics Bioinformatics and Computational Biology, Virginia Polytechnic and State University, Blacksburg, VA 24061, USA; nak3c@vt.edu; 3 Laboratory of Evolutionary Genomics of Insects, the Federal Research Center Institute of Cytology and Genetics, Siberian Branch of the Russian Academy of Sciences, 630090 Novosibirsk, Russia; 4 Laboratory of Ecology, Genetics and Environmental Protection, Tomsk State University, 634050 Tomsk, Russia; 5 Department of Cytology and Genetics, Tomsk State University, 634050 Tomsk, Russia

**Keywords:** *Anopheles*, heterochromatin, mosquito, mitotic chromosome, sex chromosome, satellite DNA, X chromosome

## Abstract

Heterochromatin is identified as a potential factor driving diversification of species. To understand the magnitude of heterochromatin variation within the *Anopheles gambiae* complex of malaria mosquitoes, we analyzed metaphase chromosomes in *An. arabiensis*, *An. coluzzii*, *An. gambiae*, *An. merus*, and *An. quadriannulatus*. Using fluorescence *in situ* hybridization (FISH) with ribosomal DNA (rDNA), a highly repetitive fraction of DNA, and heterochromatic Bacterial Artificial Chromosome (BAC) clones, we established the correspondence of pericentric heterochromatin between the metaphase and polytene X chromosomes of *An. gambiae*. We then developed chromosome idiograms and demonstrated that the X chromosomes exhibit qualitative differences in their pattern of heterochromatic bands and position of satellite DNA (satDNA) repeats among the sibling species with postzygotic isolation, *An. arabiensis*, *An. merus*, *An. quadriannulatus*, and *An. coluzzii* or *An. gambiae*. The identified differences in the size and structure of the X chromosome heterochromatin point to a possible role of repetitive DNA in speciation of mosquitoes. We found that *An. coluzzii* and *An. gambiae*, incipient species with prezygotic isolation, share variations in the relative positions of the satDNA repeats and the proximal heterochromatin band on the X chromosomes. This previously unknown genetic polymorphism in malaria mosquitoes may be caused by a differential amplification of DNA repeats or an inversion in the sex chromosome heterochromatin.

## 1. Introduction

The major malaria vector in Africa *Anopheles gambiae* has been a subject of extensive research over the past few decades. Initially described as a single species, *An. gambiae* was later subdivided into a complex of morphologically indistinguishable species by crossing experiments [1,2], fixed differences in polytene chromosome arrangement and distinct adaptations [3,4], differences in Intergenic Sequence (IGS) and Internal Transcribed Spacer (ITS) regions in the ribosomal DNA (rDNA) [5,6], and by whole-genome divergence [7,8,9]. Members of the *An. gambiae* complex differ in genetic diversity and they include major vectors, minor vector, and nonvectors [3,4,10]. The current list of the species includes *An. arabiensis, An. amharicus, An. bwambae, An. coluzzii, An. fontenillei, An. gambiae, An. merus, An. melas*, and *An. quadriannulatus* [4,7,9]. 

The majority of interspecies crosses in the *An. gambiae* complex produce fertile females and sterile males [2,11,12,13,14] in agreement with Haldane’s rule [15]. However, the most closely related species *An. coluzzii* and *An. gambiae* do not have postzygotic reproductive barriers as in laboratory conditions they readily mate and lay viable eggs that produce fertile hybrid males and females [16,17]. If the *An. gambiae* complex originated as recently as 526 thousand years ago, the *An. coluzzii* and *An. gambiae* lineages split from the common ancestor only ~61 thousand years ago [18]. The recently diverged species *An. gambiae sensu stricto* and *An. coluzzii* have been initially considered as the S and M molecular forms of *An. gambiae* [5,6] based on specific single nucleotide polymorphism (SNP) differences of ITS2 sequences [19,20]. Later studies demonstrated that the two forms also differ in genome sequence [8], gene expression [21], larval ecology [22], larval behavior in the presence of predators [23,24], adult swarming behavior [25], and adult mate recognition [17,26]. Thus, these data show evidence for ecological, behavioral, and genome-wide differentiation between *An. coluzzii* and *An. gambiae*. 

Cytogenetic analysis of the polytene chromosomes banding patterns, including fixed chromosomal inversions, is an established tool for distinguishing species of malaria mosquitoes [3,4,27,28,29]. In addition, cytogenetic analysis of mitotic chromosomes demonstrated interspecific differences in sex chromosome heterochromatin between *An. gambiae* and *An. arabiensis* (named *An. gambiae* species A and B at that time) [30]. Staining with the Hoechst fluorescent dye has shown that the presence and brightness of X-chromosome heterochromatic bands differ between the two species [31]. More recently, heterochromatin variation of mitotic Y chromosomes among species of the *An. gambiae* complex has been clearly demonstrated [32]. Intraspecific polymorphism in the X and Y chromosome heterochromatin has also been observed in both natural and laboratory populations of *An. gambiae* [30,31], but is unclear if the previously observed variations can be related to possible differences between the more recently described incipient species *An. coluzzii* and *An. gambiae s.s.* [7]. This information can be useful because vector control to be successful must consider full spectrum of genetic and phenotypic variation within vector species. Further, it has been suggested that polymorphism in sex chromosome heterochromatin may affect fertility and sexual behavior of mosquitoes [33], but the lack of understanding of heterochromatin structure and function prevent researchers from mechanistic understanding of the phenotypic effects.

Sequencing of the *An. gambiae* genome provided important information regarding its organization [34]. It has been demonstrated that repetitive DNA component represent a substantial portion in of the *An. gambiae* genome (33%) that is higher than in *Drosophila melanogaster* genome where repeats make up approximately 24% of the genome [35,36]. The majority of repetitive DNA in the *An. gambiae* genome is tightly packed in heterochromatin around the centromeres [37]. Difficulty with sequencing of heterochromatin led to underrepresentation of heterochromatic sequences in the *An. gambiae* genome assembly [34]. Moreover, the repetitive nature of these sequences poses an impediment in mapping them correctly to chromosomes. Subsequent attempts to map heterochromatic genomic scaffolds led to an addition of ~16 Mb of heterochromatin to the genome of the *An. gambiae* PEST strain [38]. Further progress was made by predicting functions of 232 heterochromatin genes [39] and by mapping genes on the heterochromatin-euchromatin boundary of the polytene chromosome map [40]. Bioinformatics analysis of so called “unknown chromosome” or ~42 Mb of unmapped sequences in *An. gambiae* PEST genome demonstrated that it has characteristics of heterochromatin [39]. Although both *An. gambiae* and *An. coluzzii* genome assemblies are now available [39,41], corresponding information of their entire heterochromatin on a chromosome map is still missing. Likewise, sequencing of five other species from the *An. gambiae* complex also excluded the majority of heterochromatic sequences from the genome assembly [9,42].

Comparison of *An. gambiae* (the former S form) and *An. coluzzii* (the former M form) genomes identified pericentromeric autosomal and X-chromosome regions of high differentiation, termed “speciation islands,” or “islands of genomic divergence” [43,44]. These regions largely correspond to pericentromeric heterochromatin. Overlaps between the heterochromatin and the islands of genomic divergence are 91% in the X chromosome, 97% in the 2L arm, and 94% in the 3L arm [39]. Other studies emphasized that the highest genomic divergence between these nascent species occurred within the 4 Mb of mapped heterochromatin on the X chromosome [8,45,46]. A recent population genomic analysis of 765 field-collected mosquitoes across Africa has demonstrated that sequences in the pericentromeric X heterochromatin show the greatest separation between *An. coluzzii* and *An. gambiae* populations on a neighbor-joining tree [47]. The study also analyzed the *An. coluzzii* and *An. gambiae* genomes for CRISPR/Cas9 target sites; it found that 5474 genes had at least one viable target after excluding target sites with nucleotide variation. Interestingly, targetable genes are spread non-uniformly across the genome, falling predominantly in pericentromeric heterochromatic regions, where levels of variation are lower [47]. However, the full spectrum of heterochromatin variation and molecular composition of the X-chromosome heterochromatin in malaria mosquitoes remains unknown. Because repetitive DNA is underreplicated [48], polytene chromosomes have limited applications in studies of heterochromatin. In comparison, mitotic chromosomes are a promising system for evolutionary studies of heterochromatin in malaria mosquitoes. 

In this study, we examined similarities and differences in heterochromatin patterns within X mitotic chromosomes among the major malaria vectors *An. gambiae*, *An. coluzzii, An. arabiensis,* minor vector *An. merus,* and zoophilic non-vector *An. quadriannulatus*. The correspondence of pericentric heterochromatin between the metaphase and polytene X chromosome was established for *An. gambiae*. We report quantitative differences in molecular organization of heterochromatin among members of the *An. gambiae* complex and highlight shared polymorphism of the heterochromatin structure among strains of the incipient species *An. gambiae* and *An. coluzzii.* The identified variation in heterochromatin is discussed with respect to the phylogenetic relationships among the species and a possible role of heterochromatin in speciation.

## 2. Materials and Methods 

### 2.1. Mosquito Strains and Colony Maintenance

Laboratory colonies examined for this study were provided by the Biodefense and Emerging Infections Research Resources Repository and included colonies of *An. gambiae* PIMPERENA (MRA-861), *An. gambiae* KISUMU1 (MRA-762), *An. gambiae* ZANU (MRA-594), *An. coluzzii* SUA2La (MRA-765), *An. coluzzii* MOPTI (MRA-763), *An. coluzzii* MALI-NIH (MRA-860), *An. arabiensis* DONGOLA 2Ra2Rb3R (MRA-1235), *An. quadriannulatus* SANGWE (MRA-1155), and *An. merus* MAF (MRA-1156). The species and strains used in this study are presented in Table S1. All mosquitoes were reared at 27 °C, with 12:12 light:dark cycle and 70% relative humidity. Authentication of the species was done using PCR diagnostics [49,50]. Larvae were fed fish food and adult mosquitoes were fed 1% sugar water. To induce oviposition, females were fed defibrinated sheep blood (Colorado Serum Co., Denver, Colorado, USA) using artificial blood feeders. To perform interspecies crosses between *An. gambiae* and *An. coluzzii*, male and female pupae were separated to ensure virginity of adult mosquitoes. We differentiated males and females at the pupal stage using sex-specific differences in the shape of their terminalia [51]. After the emergence of adults, crossing experiments were performed by combining 30 females and 15 males in a single cage. Five days after random mating, the females were fed sheep blood. Two days later, an egg dish, covered with moist filter paper to keep the eggs from drying out, was put into the cage to obtain F1 hybrid larvae for cytogenetic analyses. Any possible colony contamination during the experiment was ruled out by verifying all the strains for their respective species using intentional mismatch primers (IMP) as described elsewhere [19]. All the strains showed the expected band size, with *An. gambiae* strains around 330 bp and *An. coluzzii* strains around 460 bp.

### 2.2. Chromosome Preparation 

Preparations from early 4th instar larvae of lab colonies were made from leg and wing imaginal discs, as previously described [52]. Both male and female larvae were immobilized on ice for 10 min, and then dissected in a drop of cold, freshly prepared, hypotonic solution (0.075 M potassium chloride). After decapitating the head, the thorax was opened using dissecting scissors (Fine Science Tools, Foster City, CA, USA), followed by removal of the gut and fat from the body. A fresh drop of hypotonic solution was added to the preparation for 10 min, followed by fixation in a drop of modified Carnoy’s solution (ethanol:glacial acetic acid, 3:1) for 1 min. Next, a drop of freshly prepared 50% propionic acid was added, and the imaginal discs were covered with a 22 × 22 mm coverslip. After 5 min, the preparations were squashed using the flat rubber end of a pencil and dipped in liquid nitrogen until the bubbling stopped. Coverslips were removed using a sharp blade and slides were transferred to cold 50% ethanol and stored at −20 °C. After 2 h, slides were dehydrated in a series of 70%, 80%, and 100% ethanol. Preparations with the highest number of metaphase plates were chosen for *in situ* hybridization. 

### 2.3. C_0_t DNA Preparation and DNA Probe Labeling 

To identify the position of the centromere in chromosome X, we prepared the repetitive DNA fraction using a previously described method [52]. Genomic DNA was isolated from 500 g of non-bloodfed adult *An. coluzzii* MOPTI mosquitoes using a Qiagen Blood and Cell culture DNA Maxi kit (Qiagen, Hilden, Germany). Isolated C_0_t1 repetitive fraction was precipitated with isopropanol and labelled by nick-translation in 50 μl, containing 1 μg DNA, 0.05 mM each of unlabeled dATP, dCTP, and dGTP, and 0.015 mM of dTTP, 1 μl of fluorescein-dUTP, 0.05 mg/ml of BSA, 5 μl of 10× nick-translation buffer, 20 U of DNA-polymerase I, and 0.0012 U of DNase, at 15 °C for 2.5 h. Primers for 18S rDNA and satDNA repeats from *An. gambiae* AgY53A, AgY477-AgY53B, AgY477, and AgY53C were obtained, as previously described [14,53] (Table S2). An Immomix PCR kit (Bioline, Inc., Taunton, MA, USA) was used to label satellites by incorporating Cy3 and Cy5 fluorescently labeled nucleotides (Enzo Life Sciences, Inc., Farmingdale, NY, USA) directly into the PCR reaction. Each 25 µl PCR mix consisted of 35–40 ng genomic DNA, 0.3 U Taq polymerase, 1× PCR buffer, 200 µM each of dATP, dCTP, and dGTP, and 65 µM dTTP, and 0.5 µl Cy3-dUTP or 0.5 µl Cy5-dUTP (Enzo Life Sciences, Inc., Farmingdale, NY, USA). Thermocycling was performed using ImmoMix TM (Bioline Inc., Taunton, MA, USA) beginning with a 95 °C incubation for 10 min followed by 35 cycles of 95 °C for 30 sec, 52 °C for 30 sec, 72 °C for 45 sec, 72 °C for 5 min, and a final hold at 4 °C. Bacterial Artificial Chromosome (BAC) clones 05F01 (GenBank accession: AL142298, AL142299), 179F22 (GenBank accession: BH373300, BH373306), and 01K23 (GenBank accession: AL607293, AL607294) [34] were labelled by nick-translation in 50 μl, containing 1 μg DNA, 0.05 mM each of unlabeled dATP, dCTP, and dGTP and 0.015 mM of dTTP, 1 μl of Cy3-dUTP (or another fluorochrome), 0.05 mg/ml of BSA, 5 μl of 10× nick-translation buffer, 20 U of DNA-polymerase I, and 0.0012 U of DNase at 15 °C for 2.5 h.

### 2.4. Fluorescence in situ Hybridization (FISH)

Suitable slides with >10 metaphase plates were selected for FISH, which was performed as previously described [52,54]. Briefly, slides with good preparations were treated with 0.1 mg/ml RNase at 37 °C for 30 min. After washing twice with 2× saline-sodium citrate (SSC) for 5 min, slides were digested with 0.01% pepsin and 0.037% HCl solution for 5 min at 37 °C. After washing slides twice in 1× phosphate-buffered saline (PBS) for 5 min at room temperature, preparations were fixed in 3.7% formaldehyde for 10 min at RT. Slides were then washed in 1× PBS and dehydrated in a series of 70%, 80%, and 100% ethanol for 5 min at RT. Then, 10 μl of probes were mixed, added to the preparations, and incubated overnight at 37 °C. After washing slides in 1× SSC at 60 °C for 5 min, 4× SSC/NP40 solution at 37 °C for 10 min, and 1× PBS for 5 min at room temperature (RT), preparations were counterstained with a DAPI-antifade solution (Life Technologies, Carlsbad, CA, USA) and kept in the dark for at least 2 h before visualization with a fluorescence microscope.

### 2.5. Image Acquisition and Chromosome Measurements

Chromosome slide preparations were viewed with an Olympus BX61 fluorescence microscope (Olympus, Tokyo, Japan) using BioView software (BioView Inc., Billerica, MA, USA) at 1000× magnification. For idiogram development, metaphase plates were measured and analyzed from 5 larvae per strain. A total of 40 metaphase plates were used to measure chromosome lengths in *An. coluzzii*. For sibling species *An. arabiensis*, *An. quadriannulatus*, and *An. merus*, 11–13 metaphase plates were used to measure chromosome lengths (Table S3). Chromosome images were inverted using Adobe Photoshop CS6 (Adobe Inc., San Jose, CA, USA) and measured using the ruler tool at 1000× magnification. A statistical Tukey’s test or a nonparametric Kruskal–Wallis rank sum test followed by a Dunn’s test were performed to compare chromosome length between the species using JMP13 software (SAS Institute Inc., Cary, NC, USA) or R software (RStudio, Boston, MA, USA). Fluorescence intensities of proximal and distal X chromosome heterochromatin bands were measured using Adobe Photoshop CS6 (Adobe Inc., San Jose, CA, USA) and compared between three strains of *An. gambiae* and three strains of *An. coluzzii*. About forty measurements for each band were taken from forty sister chromatids of twenty X chromosomes for each strain. Pairwise comparisons between strains were performed using Student’s t-test.

### 2.6. Quantative Analysis of Fluorescent Signal Positions

Chromosomal positions of satDNA AgY477–AgY53B and AgY477, and the proximal DAPI band were identified and compared in three strains of *An. gambiae* and three strains of *An. coluzzii*. A custom MATLAB script [55] was written and used to measure the position of fluorescence peaks in individual channels [56]. The program was automated to measure the position of each satDNA and DAPI peak along the length of the X chromosome. The output provided the maximum likelihood of a combination of probe positions. The results were compared within and between species. The MATLAB program consisted of two user-guided steps followed by a third step of automated analysis. In the first step, sex chromosomes were identified by the user on mitotic slide preparations from *An. gambiae* and *An. coluzzii* strains. In the second step, the boundary of each identified sex chromosome was traced by a trained user. In an automated step, the pattern of heterochromatin and satDNA fluorescence was averaged longitudinally along the user-defined boundary. Program output displayed fluorescence intensity graphically from chromosome centromere to telomere. Peak fluorescence intensity was used to automatically infer the order of the heterochromatin and satDNA probes. User guidance was deliberately introduced to eliminate the pitfalls of full automation. In particular, user guidance was critical for accurate identification of sex chromosomes in the imaged mitotic slide preparations. Longitudinal averaging of fluorescence intensity during the program’s automated analysis was robust to the boundary of each chromosome traced by the user. 

## 3. Results

### 3.1. Correspondence Between Heterochromatin of the Polytene and Mitotic X Chromosome in Anopheles gambiae

**Figure 1:**
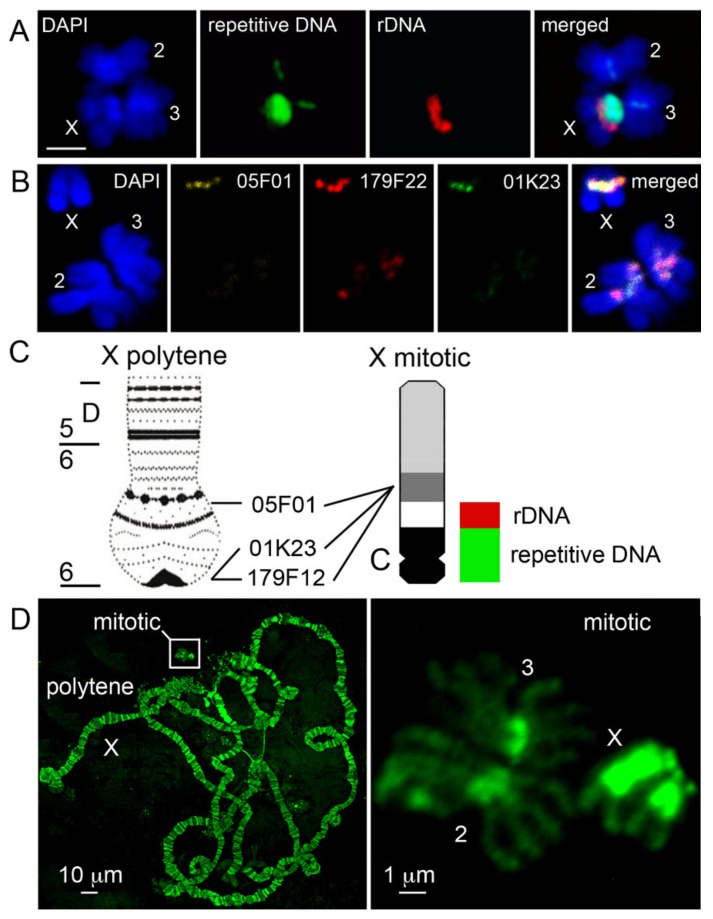
Comparison of the X heterochromatin structure between mitotic and polytene chromosomes. (**A**) Multicolor fluorescence *in situ* hybridization (FISH) of a C_0_t1 fraction of repetitive DNA and 18S rDNA with mitotic chromosomes of *An. gambiae* PIMPERENA; scale bar is 2 µm. (**B**) FISH mapping of BAC clones 05F01 (yellow), 179F22 (red), and 01K23 (green) to the distal heterochromatin block on the X chromosome of the *An. coluzzii* MOPTI strain. Chromosomes are counter-stained with DAPI. (**C**) Relative positions of heterochromatic blocks and DNA probes of the polytene and mitotic X chromosome idiograms. rDNA = 18S rDNA probe. Repetitive DNA = C_0_t1 fraction of repetitive DNA. C = putative centromere. (**D**) Relative sizes of mitotic and polytene chromosomes in the KISUMU strain of *An. gambiae.* Chromosomes are counter-stained with YOYO-1. Polytene chromosome physical map is from [38].

Using FISH with a C_0_t1 fraction of repetitive DNA, 18S rDNA, and heterochromatic BAC clones, we attempted to establish the correspondence of pericentric heterochromatin between the metaphase and polytene X chromosome in *An. gambiae*. The most repetitive C_0_t1 DNA fraction should presumably represent the centromeric satellites of the mitotic chromosomes corresponding to the most proximal regions of polytene arms. C_0_t analysis is the process of renaturation of single stranded DNA to its complementary sequence [57]. The analysis is based on the observation that the more repetitive DNA sequences require a shorter time to re-anneal following denaturation. DNA fractions with C_0_t1 values equal to 1.0 × 10^−4^–1.0 × 10^−1^ are considered as highly repetitive DNA fractions [57]. We expected C_0_t1 to map to narrow regions corresponding to centromeres in mitotic chromosomes. While that was the case for autosomes, in X chromosomes the C_0_t1 DNA probe localized to a wide region, including the entire proximal heterochromatin band adjacent to the rDNA locus (Figure 1A). Thus, hybridization of the C_0_t1 DNA fraction could be indicative of the centromere positions in autosomes, but we could not precisely determine the location of the centromere on the X chromosome in *An. gambiae*. We mapped three heterochromatic BAC clones, 05F01, 179F22, and 01K23, to the distal heterochromatin band of mitotic chromosomes in *An. coluzzii* (Figure 1B). According to the *An. gambiae* PEST genome assembly [38]*,* these BAC clones are located in the most proximal heterochromatic region 6 in the X polytene chromosome map (Figure 1C). BAC clone 05F01 is mapped to scaffold AAAB01008973 (X: 20,698,730–20,818,594), BAC clone 01K23 is mapped to scaffold AAAB01008967 (X: 23,512,846–23569864), and BAC clone 179F22 is mapped to scaffold AAAB01008976 (X: 23,930,050–24,043,835) [38]. These results demonstrate that the assembled euchromatic part of the X chromosome occupies no more than half of the total X chromosome length (Figure 1C). Although polytene chromosomes are significantly larger than mitotic chromosomes, heterochromatin is under-replicated [48] and represented by diffuse structures without clear banding patterns [39], while heterochromatin in mitotic chromosomes is much more prevalent and detailed (Figure 1D). Because the *An. gambiae* PEST X chromosome genome assembly ends at the coordinate 24,393,108, our data indicate that the assembled heterochromatin largely corresponds to the distal heterochromatic band of the mitotic X chromosome. Only few copies of 18S rDNA sequences are found in the assembled X chromosome. Thus, most of the rDNA repeats and more proximal regions of the X chromosome heterochromatin are absent from the mapped genome assembly.

### 3.2. Variation in Heterochromatin Morphology Among Sibling Species of the An. gambiae Complex

**Figure 2:**
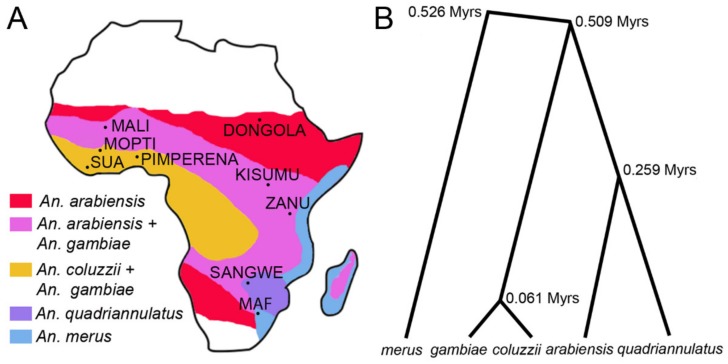
Geographical distribution of species and phylogeny of the *An. gambiae* complex. (**A**) A map of Africa with approximal distribution of species and places of origin of the laboratory strains. (**B**) Species phylogeny based on the X chromosome genomic sequences (redrawn from [18]). Times of species divergence in million years (Myrs) are shown at the tree nodes.

We analyzed the heterochromatin morphology in species of the *An. gambiae* complex originating from different parts of Africa (Figure 2A) and diverging from each other from ~61,000 to ~526,000 years ago [18] (Figure 2B).

**Figure 3:**
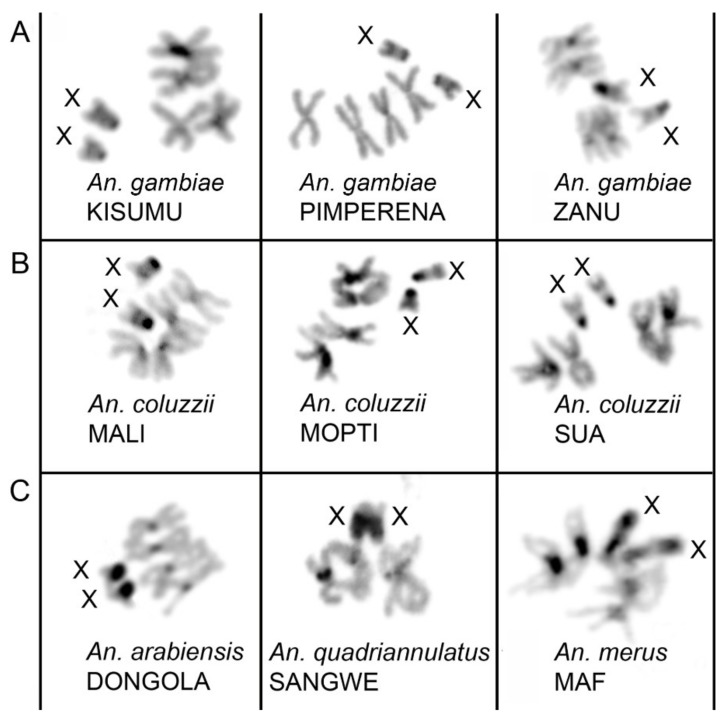
Metaphase karyotypes of females in species from the *An. gambiae* complex. (**A**) *An. gambiae* strains. (**B**) *An. coluzzii* strains. (**C**) *An. arabiensis*, *An. quadriannulatus*, and *An. merus* strains. Black and dark gray blocks correspond to compact and diffuse heterochromatin, respectively. X chromosomes are labeled. Species names are indicated in italics and strain names are capitalized.

We studied mid-metaphase chromosomes obtained from imaginal discs of the 4th instar larvae of *An. gambiae*, *An. coluzzii*, *An. arabiensis, An. merus,* and *An. quadriannulatus*. Chromosomes at this stage provide reproducible heterochromatin patterns verified across multiple individuals. DAPI, a counter-stain that preferentially stains AT-rich DNA, was utilized to augment the natural banding patterns resulting from heterochromatin variation between and within species. To simplify the comparison, all fluorescence images were inverted, and converted to gray-scale images (Figure 3). The number and size of the X chromosome heterochromatin bands varied across the sibling species. Differences in the X chromosome heterochromatin pattern were clearly visible between all species with postzygotic reproductive isolation (Figure 3). Two relatively small heterochromatin bands can be seen in *An. gambiae* and *An. coluzzii* (Figure 3A,B). The KISUMU strain of *An. gambiae* often shows polymorphism in the size of the heterochromatin between different X chromosomes, even within one individual (Figure 1D and Figure 3A) Extended heterochromatin is observed in X chromosomes of *An. arabiensis, An. quadriannulatus*, and *An. merus* (Figure 3C). The pattern of fluorescence intensity of the heterochromatic bands differs among species as well. *An. gambiae* and *An. coluzzii* possess a dense heterochromatin block on the end proximal to the putative centromere. This is followed by a dull fluorescence area to which the 18S rDNA locus is mapped. A light distal band is present immediately next to the rDNA locus, marking the assembled heterochromatin in the current *An. gambiae* PEST genome assembly [38]. A large dark heterochromatin band is visible in the *An. arabiensis* X chromosome. Our results for *An. gambiae* and *An. arabiensis* corroborated with those for the natural populations of these species in a previous study [38]. In contrast, *An. quadriannulatus*, a zoophilic non-vector species of the *An. gambiae* complex, has one light and two dark heterochromatin bands. The longest X chromosome was found in *An. merus*, the marshy saltwater resident of the *An. gambiae* complex. The *An. merus* X chromosome contains three large heterochromatin blocks and a large rDNA locus making it comparable in length to the autosomes.

### 3.3. Molecular Variation of Heterochromatin Among Species of the An. gambiae Complex

**Figure 4:**
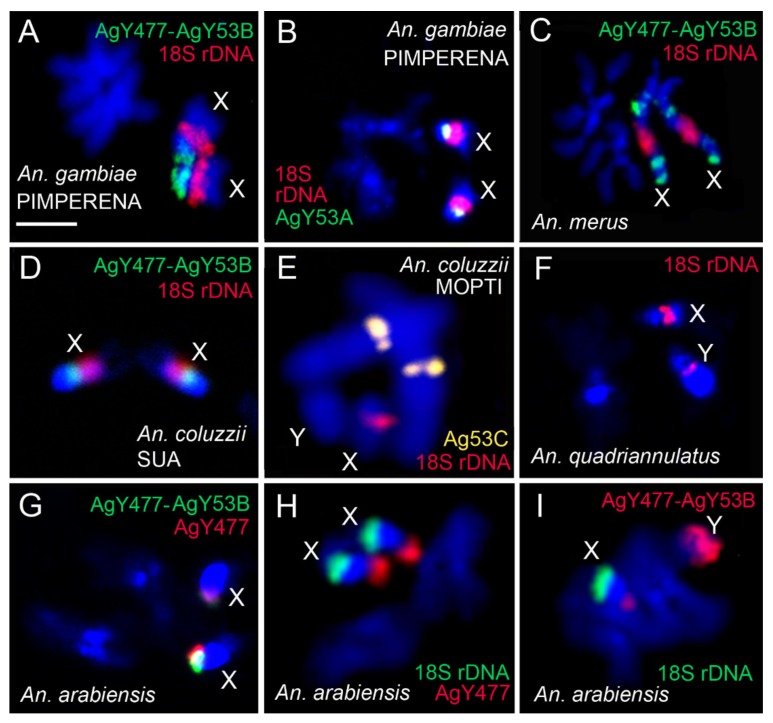
Mapping of repetitive DNA elements to mitotic chromosomes of species from the *An. gambiae* complex. (**A**) FISH of AgY477–AgY53B (green) and 18S rDNA (red) in *An. gambiae* PIMPERENA. (**B**) FISH of satellite AgY53A (green) and 18S rDNA (red) in *An. gambiae* PIMPERENA. (**C**) FISH of AgY477–AgY53B (green) and 18D rDNA (red) in *An. merus*. (**D**) FISH of AgY477–AgY53B (green) and 18S rDNA (red) in *An. coluzzii* SUA. (**E**) FISH of satellite Ag53C (yellow) and 18D rDNA (red) in *An. coluzzii* MOPTI. (**F**) FISH of 18S rDNA (red) in *An. quadriannulatus*. (**G**) FISH of satellite AgY477 (red) and AgY477–AgY53B (green) in *An. arabiensis*. (**H**) FISH of satellite AgY477 (red) and 18D rDNA (green) in *An. arabiensis*. (**I**) FISH of 18S rDNA (green) and AgY477–AgY53B (red) in *An. arabiensis*. Scale bar = 2 µm.

We analyzed similarities and differences in the molecular content of the heterochromatin among sibling species. We performed FISH with 18S rDNA and previously identified satDNA sequences AgY53A, Ag53C, AgY477, and the genomic fragment AgY477–AgY53B that contains partial sequences of satellites AgY477 and AgY53B as well as a junction region [53] (Table S2). In *An. gambiae* PIMPERENA, satellites AgY53A and AgY477–AgY53B hybridized to single locations in the tip and base of the X chromosome proximal heterochromatin band, respectively (Figure 4A,B). This is in sharp contrast to *An. merus*, where AgY477–AgY53B hybridized with four distinct regions of the X chromosome heterochromatin (Figure 4C). Interestingly, AgY477–AgY53B hybridized differently in *An. coluzzii* SUA compared with *An. gambiae* PIMPERENA; AgY477–AgY53B is found in the base of the X chromosome proximal heterochromatin band of *An. coluzzii* SUA (Figure 4D). Satellite AgY477 has also been mapped to the base of the X chromosome proximal heterochromatin band of *An. gambiae* ZANU [32]. As described in the following results section, localization of these satellites represents shared variation among strains of *An. gambiae* and *An. coluzzii.* Unlike AgY53A and AgY477–AgY53B, satellite Ag53C is not sex chromosome-specific [53]; it hybridized with pericentromeric regions of autosomes in *An. coluzzii* MOPTI (Figure 4E). The 18S rDNA probe was mapped to the space between two dense heterochromatic bands on the X chromosome in *An. quadriannulatus* (Figure 4F). Previously, it was demonstrated that the AgY477–AgY53B sequence is immediately adjacent to the rDNA locus on the X chromosome of *An. quadriannulatus* [32]. Both AgY477–AgY53B and AgY477 co-localize at the very tip of the X chromosome outside the major dense heterochromatin band in *An. arabiensis* (Figure 4G). Hybridization of each of these satellites, together with 18S rDNA, demonstrated that, unlike in *An. gambiae* or *An. coluzzii*, AgY477 (Figure 4H) and AgY477–AgY53B (Figure 4I) are separated from the rDNA locus by the large band of dense heterochromatin in *An. arabiensis.*

### 3.4. Shared Heterochromatin Variation Between An. coluzzii and An. gambiae

To visualize the rDNA locus, we performed FISH of the 18S rDNA probe with mitotic chromosomes. Within all strains of *An. gambiae* and *An. coluzzii*, the rDNA locus was mapped between the proximal and distal heterochromatin bands (Figure S1). We noticed variation in the size of the rDNA locus both among the strains of *An. gambiae* and *An. coluzzii* and between homologous chromosomes within individual mosquitoes. The intra-strain polymorphism in the size of the rDNA locus was especially obvious in KISUMU, ZANU, and MOPTI. This polymorphism may suggest variation in the rDNA copy number.

**Figure 5:**
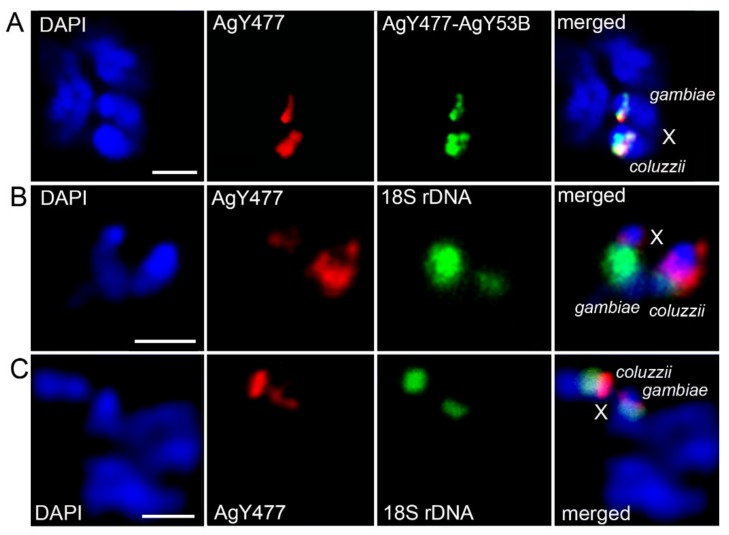
Variation in the pattern of heterochromatin blocks, rDNA loci, and satDNA location on the X chromosomes in F1 female hybrids between *An. gambiae* and *An. coluzzii.* (**A**) FISH of AgY477–AgY53B (green) and AgY477 (red) in F1 ♀*An. gambiae* PIMPERENA × ♂*An. coluzzii* MOPTI. (**B**) FISH of AgY477 (red) and 18S rDNA (green) in F1 ♀*An. gambiae* ZANU × ♂*An. coluzzii* MALI. (**C**) FISH of AgY477 (red) and 18S rDNA (green) in F1 ♀*An. coluzzii* MOPTI × ♂*An. gambiae* KISUMU. Scale bar = 2 µm.

To better resolve the relative satDNA positions among multiple mosquito strains, we generated F1 hybrids between laboratory strains of *An. gambiae* and *An. coluzzii*. F1 hybrid larvae resulting from the *An. gambiae* × *An. coluzzii* crosses were analyzed for heterochromatin morphology and satDNA location. Qualitative and quantitative differences in the pattern of heterochromatin blocks, rDNA loci, and satDNA location were clearly observed between homoeologous X chromosomes within the F1 hybrids. Different positions and sizes of the AgY477–AgY53B and AgY477 FISH signals with respect to the proximal heterochromatin band can be seen between homoeologous X chromosomes in F1 ♀*An. gambiae* PIMPERENA × ♂*An. coluzzii* MOPTI (Figure 5A). Different sizes of the proximal heterochromatin band and the rDNA locus can be seen between homoeologous X chromosomes in F1 ♀*An. gambiae* ZANU × ♂*An. coluzzii* MALI (Figure 5B). Also, different sizes of the hybridization signals from AgY477 and 18S rDNA can be seen between homologous X chromosomes in F1 ♀*An. coluzzii* MOPTI × ♂*An. gambiae* KISUMU (Figure 5C).

**Figure 6:**
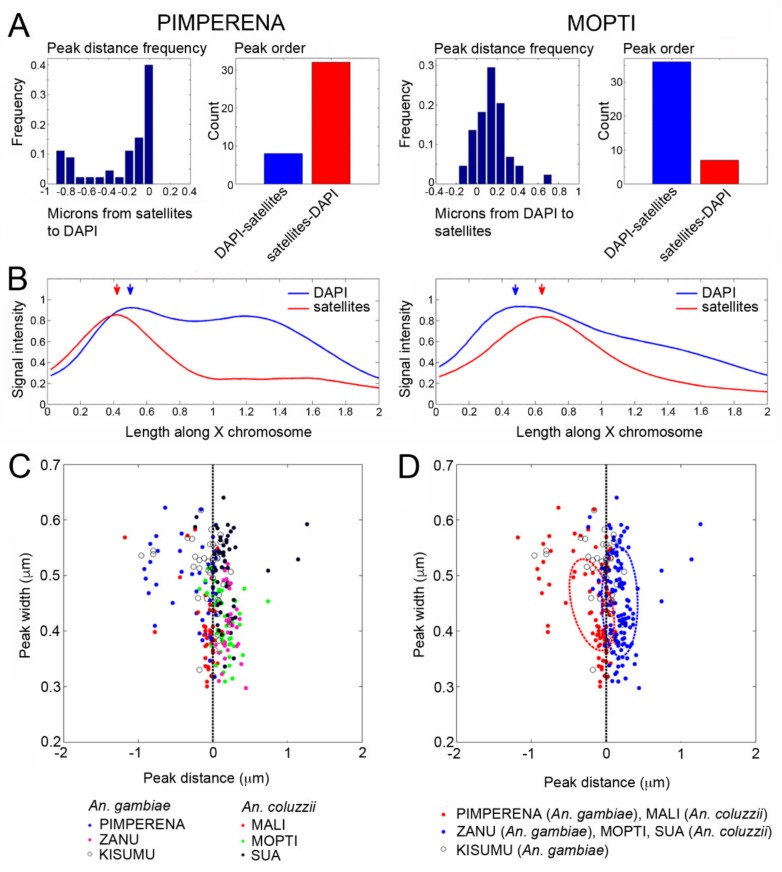
Polymorphism of the X heterochromatin structure among the *An. gambiae* and *An. coluzzii* strains. (**A**) Distance and order frequency of FISH signal and DAPI band intensity peaks in *An. gambiae* PIMPERENA and *An. coluzzii* MOPTI. (**B**) Distribution patterns of signal intensities for satellites and DAPI along the X chromosomes in *An. gambiae* PIMPERENA and *An. coluzzii* MOPTI. (**C**) Relative positions of satellite peak distance and width with respect to the proximal heterochromatin band for six strains of *An. gambiae* and *An. coluzzii*. (**D**) Clustering of the *An. gambiae* and *An. coluzzii* strains based on the relative positions of satellite peak distance and width with respect to the proximal heterochromatin band.

Using a custom MATLAB script [56], the position of the satDNA repeats with respect to the proximal heterochromatin band was mapped on the X chromosomes in *An. gambiae* and *An. coluzzii* strains. Because sequences of AgY477–AgY53B and AgY477 overlap, it is technically unfeasible to distinguish between the signals when mapping their distance from the proximal heterochromatin band. Therefore, we combined the fluorescence signals of AgY477–AgY53B and AgY477 and mapped them with respect to the fluorescence DAPI peak of the heterochromatin. The advantages of our automated analysis are twofold. First, fluorescent signals in each image possess a degree of overlap, which masks the order of fluorescent peaks from centromere to telomere; however, the order of peaks is easily detected using our automated analysis. Second, observation alone is insufficient to determine the distance between fluorescent peaks, which are generally separated by fewer than 10 pixels. Determining the distance between nearby peaks is made tractable in our pipeline; however, even this computational approach remains limited by microscopic resolution. Images of the mitotic sex chromosomes were captured using 1000× magnification; neighboring pixels at this resolution represent a distance of 0.05 μm. Thus, peaks spaced closer than 0.05 μm were prone to falsely inverted order. Nonetheless, peak orders were generally resolvable by aggregating multiple images averaged for multiple individuals of each strain. We measured the distance from satellites to the fluorescence DAPI peak and the linear order of the peaks. For example, the order of the satDNA and DAPI peaks is switched between *An. gambiae* PIMPERENA and *An. coluzzii* MOPTI (Figure 6A). Distribution patterns of signal intensities for satellites and DAPI along the X chromosomes also differ between *An. gambiae* PIMPERENA and *An. coluzzii* MOPTI (Figure 6B). Our mapping results revealed that the strains differed in the pattern of satDNA and DAPI peak relative positions and distance on the X chromosome (Figure 6C). Clustering analysis showed that *An. gambiae* PIMPERENA and *An. coluzzii* MALI depicted the same pattern of satDNA, followed by the DAPI band, while *An. coluzzii* MOPTI, *An. coluzzii* SUA, and *An. gambiae* ZANU clustered together showing the DAPI peak followed by the satDNA locus (Figure 6D). Interestingly, *An. gambiae* KISUMU had an almost 1:1 ratio of the direct and the reverse satDNA-DAPI band order indicating that this strain is maintaining high polymorphism in their X chromosome heterochromatin. Thus, our data shows the dynamism of the heterochromatin at the molecular level in the important malaria vectors. We found no evidence for clustering the strains by species, indicating that this heterochromatin variation is shared between *An. coluzzii* and *An. gambiae* either as ancestral polymorphism or by ongoing hybridization between the incipient species.

We also compared the average fluorescence intensities of the proximal and distal X chromosome heterochromatin between three strains of *An. gambiae* and three strains of *An. coluzzii*. A statistical analysis, using Student’s t-test, revealed no specific pattern when strains were compared with respect to the species. Most strains were significantly different from each other in their fluorescence intensities of the proximal band (*P* < 0.05), except for SUA-PIMPERENA (*P* = 0.06), SUA-ZANU (*P* = 0.16), ZANU-PIMPERENA (*P* = 0.61), and MALI-KISUMU (*P* = 0.74). MOPTI was significantly different from every other strain (Figure S2A). Comparison of fluorescence intensities of the distal heterochromatin band revealed a similar pattern for pairwise comparison between strains of both species. Most strains were significantly different from each other (*P* < 0.05), except for PIMPERENA-KISUMU (*P* = 0.07), ZANU-MALI (*P* = 0.11), and KISUMU-SUA (*P* = 0.89) (Figure S2B). Overall, the fluorescence intensities of both X chromosome heterochromatin bands vary among the strains, regardless of their species identity.

### 3.5. X Chromosome Idiograms for Species of the An. gambiae Complex

**Figure 7:**
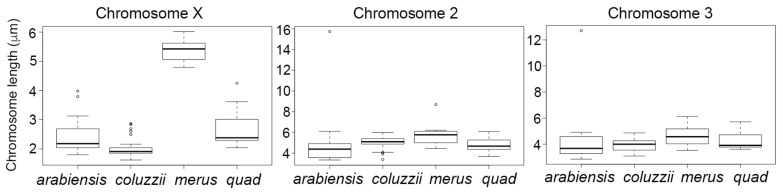
Lengths of the metaphase chromosomes in species from the *An. gambiae* complex.

To construct idiograms, we measured the lengths of the chromosomes (Table S3) and conducted a statistical pairwise Tukey’s test between the sibling species. Our analysis revealed that the total X chromosome lengths were significantly different for all species pairs, except between *An. quadriannulatus* and *An. arabiensis* (*P* = 0.64). Due to the possibility that the X chromosome length data violated the assumption of equal variance, we repeated statistical analyses using a nonparametric Kruskal–Wallis rank sum test followed by a Dunn’s test. These analyses resulted in qualitatively identical results (Figure 7, Table S4). The polytene X chromosomes in all these species are similar in length; hence, the difference in mitotic X chromosomes can be attributed to the variation in heterochromatin.

We also conducted a statistical pairwise Tukey’s test for autosomes between the sibling species. We found that, unlike the X chromosome, the autosomal lengths do not significantly differ between the species (Figure 7, Table S5). This finding demonstrates the contrasting patterns of the sex chromosome and autosome evolution and highlights the rapids evolutionary changes in the X chromosome heterochromatin. 

**Figure 8:**
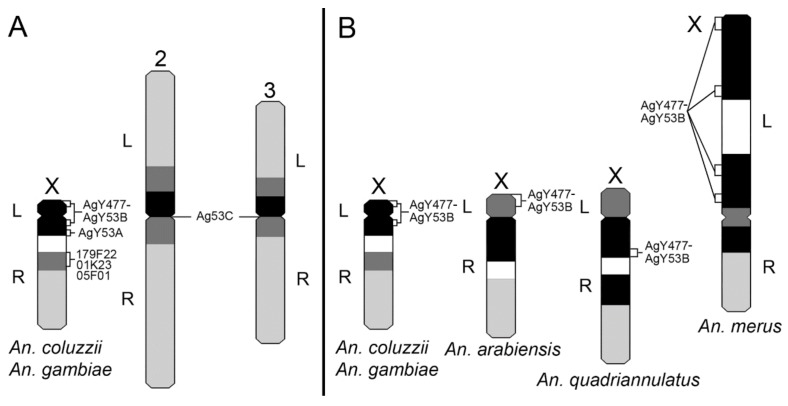
Idiograms of metaphase karyotypes of species from the *An. gambiae* complex. (**A**) An idiogram of metaphase karyotypes of *An. gambiae* and *An. coluzzii*. (**B**) Comparison of the X chromosome idiograms among species from the *An. gambiae* complex. Left and right arms are labeled with L and R, respectively. Black bands correspond to condensed heterochromatin. Dark gray bands correspond to diffuse heterochromatin. White areas represent the rDNA locus. Light gray areas show euchromatin. The area of constriction represents the putative centromere. Mapping of the BAC clones and satDNA repeats AgY53A and AgY53C were performed only with *An. coluzzii* or *An. gambiae*. Two different positions of AgY477–AgY53B on the X chromosome of *An. gambiae* and *An. coluzzii* represent polymorphism among strains.

Based on mitotic chromosome measurements, banding patterns, and repeat mapping, we constructed X chromosome idiograms for *An. gambiae, An. coluzzii, An. arabiensis, An. quadriannulatus,* and *An. merus*. FISH with the genomic fragment AgY477–AgY53B revealed its multiple locations in the X chromosome heterochromatin bands in *An. merus* but a single location in the pericentric heterochromatin in other sibling species. These features were placed on respective idiograms for easy comparison of molecular features within heterochromatin among species (Figure 8). The identified differences in the X chromosome size and heterochromatin structure are consistent with *An. gambiae, An. coluzzii, An. arabiensis, An. quadriannulatus* grouping together while *An. merus* being a separate lineage in the species phylogeny [18] (Figure 2B). The *An. merus* X chromosome has the largest amount of heterochromatin among the X chromosomes of sibling species. Moreover, *An. merus* is the only species in this study with the rDNA locus located in the L arm of the X chromosome suggesting a fixed pericentric inversion that differentiate *An. merus* from the other species. The developed idiograms will facilitate future studies of genetic variation in repeat-rich heterochromatic regions of malaria mosquitoes from the *An. gambiae* complex.

## 4. Discussion

Mosquitoes, with their small number of diploid chromosomes, 2*n* = 6, present a convenient model to investigate the molecular organization of their genomes and chromosome evolution [58]. Since polytene chromosomes are well-developed in *Anopheles* mosquitoes, they are often preferred over mitotic chromosomes for gene mapping [40,59]. Analysis of polytene chromosomes has been useful for identifying genomic variations in euchromatin. Comparative genome mapping within the *Anopheles* genus has demonstrated that the euchromatic portion of the X chromosome is the fastest evolving by genomic rearrangements [42,60]. However, under-replication of heterochromatin in polytene chromosomes makes them less suitable for mapping repetitive elements of the genome. In this study, we utilized metaphase mitotic chromosomes for repeat mapping and for studying heterochromatin variation among species in the *An. gambiae* complex. Since the genome of *An. gambiae* was first published [34], polytene chromosomes have been used to improve the heterochromatin assembly and to characterize its genetic content [38,39,40]. Here, we established the correspondence of pericentric heterochromatin between the metaphase and polytene X chromosome of *An. gambiae*, and demonstrated that at least half of the *An. gambiae* X chromosome length is heterochromatic. Future long-read sequencing and assembly of the heterochromatin in malaria mosquitoes may yield important insights into structural and functional organization of the sex chromosomes, as demonstrated for *Drosophila* [61].

Our comparative analysis of the X chromosome pericentric heterochromatin identified qualitative differences among sibling species, *An. arabiensis*, *An. merus, An. quadriannulatus,* and *An. coluzzii* or *An. gambiae*, with postzygotic isolation. The identified fixed variations included the number and pattern of heterochromatic bands and chromosomal location of the satDNA repeats. A common emerging theme from studies performed in *Drosophila* suggests that heterochromatin plays an important role in hybrid incompatibility [62,63,64,65,66]. Rapidly evolving heterochromatic repeats and heterochromatin-binding proteins are considered major players in the evolution of intrinsic reproductive isolation. Reproductively isolated *Drosophila* species have different satDNA content and localization within heterochromatin [64,67]. Evolutionary forces lead to a great variation in the copy number and types of heterochromatin repeats between closely related species. As repetitive DNA elements replicate, mechanisms such as unequal crossover, rolling circle replication, and segmental duplication become sources of genome evolution [68,69]. Thus, our study supports observations from other organisms that sex chromosomes have a propensity to accumulate species-specific differences in heterochromatin [70].

SatDNA repeats are seen as prime candidates to trigger genome instability in interspecies hybrids [71]. They have been implicated in disruption of chromosome pairing, abnormal heterochromatin packaging, and selfish post-meiotic drive substitutions in *Drosophila* hybrids [67]. Disruption of chromosome pairing and synapsis is observed frequently in interspecies hybrids of various organisms. Interestingly, sex chromosomes more often than autosomes drive speciation and hybrid incompatibilities [70]. For example, pairing of X and Y chromosomes is more adversely affected than that of autosomes during the prophase I in male hybrids between Campbell’s dwarf hamster and the Djungarian hamster [72]. A recent work has clarified that the autosomes of male hybrids between these hamster species undergo pairing and recombination normally as their parental forms do, but the heterochromatic arms of the X and Y chromosomes show a high frequency of asynapsis and recombination failure [73]. It was proposed that asynapsis of heterospecific chromosomes in prophase I may provide a recurrently evolving trigger for the meiotic arrest of interspecific F1 hybrids of mice [74,75]. Our recent study showed that meiotic failures in sterile F1 ♀*A. merus* × ♂*A. coluzzii* hybrids are accompanied by the disruption of sex chromosome pairing and insufficient chromosome condensation. These cytogenetic abnormalities lead to a premature equational division and formation of diploid immotile sperm [14]. Demonstrated here, qualitative inter-species differences in heterochromatin suggest that they may be responsible for incompatibilities of sibling species from the *An. gambiae* complex.

Unlike species with postzygotic isolation, *An. coluzzii* and *An. gambiae* do not show fixed differences in the pattern of the X chromosome pericentric heterochromatin. An early study observed an intra-specific polymorphism of the X chromosome heterochromatin among lab strains of *An. gambiae* [31]. The described polymorphism was based on the Hoechst staining of the heterochromatin. Here, we studied the pattern of heterochromatic bands or location of satDNA in multiple strains of *An. coluzzii* and *An. gambiae*, incipient species with prezygotic isolation. We found that, although the strains may differ from each other, the species do share variation in the relative positions of the fluorescence intensity peaks for the satDNA repeats and the proximal DAPI-positive heterochromatin band of the X chromosomes. The mechanism behind this inversion-like genetic polymorphism in malaria mosquitoes could be a rapid differential amplification of satellite repeats or actual structural rearrangement in the sex chromosome heterochromatin. It is possible that further evolution of heterochromatin in *An. coluzzii* and *An. gambiae* will contribute to higher genetic differentiation, and will eventually lead to postzygotic reproductive isolation between some populations of these important disease vectors. Extensive studies of *Drosophila* species show that heterochromatin, in general, and satellite DNA, in particular, can be a source of tremendous genetic variation [76,77,78,79,80]. Heterochromatin can also modulate differential gene expression and cause variable phenotypes, including differences in immune response [81,82]. It is possible that changes in repetitive sequences can have fitness consequences upon which selection will act. A population genomics study in *D. melanogaster* found that satDNA repeats often show population differentiation, but the population structure inferred from overall satellite quantities does not recapitulate the expected population relationships based on the demographic history of this species [79]. Moreover, some satellites have likely been involved in antagonistic interactions, as inferred from negative correlations among them. 

The rDNA locus is immediately adjacent to the heterochromatin bands of the X chromosome. Its variation in size, among and within species, suggests polymorphism of the rDNA copy number. Natural polymorphism in the number of rDNA copies has been recorded in vertebrate and invertebrate animals, both among and within species [83]. Associations between the heterochromatin or rDNA variation and organismal phenotypes have also been demonstrated. A study in *Drosophila* showed that heterochromatin formation prolongs lifespan and controls ribosomal RNA synthesis [84]. Another study demonstrated that rDNA copy number decreases during aging, and that this age-dependent decrease in rDNA copy number is transgenerationally heritable [85]. In humans, the number of rDNA repeats can vary from 250 to 670 copies per diploid genome [86], and the changes in rDNA copy number can affect genome-wide gene expression [87]. Natural variation in rDNA and heterochromatin may contribute to genome evolution, formation of reproductive barriers, and eventually to diversification of species [88,89,90]. Genomics studies of the heterochromatin in natural populations of *An. coluzzii* and *An. gambiae* can determine the actual levels of polymorphism and divergence in satDNA and rDNA copy number variation. These studies may also identify heterochromatin variations that correlate with specific environmental adaptations or behaviors of the malaria vectors.

## 5. Conclusions

We discovered a new type of shared cytogenetic polymorphism in the incipient species, *An. gambiae* and *An. coluzzii*—an inversion of the satDNA location with respect to the proximal heterochromatin band. This finding suggests mechanisms of rapid differentiation of the sex chromosome heterochromatin during evolution: genomic rearrangement or differential amplification of heterochromatic repeats. Future genome sequencing using long-read technologies will be crucial for determining the precise nature of the observed polymorphism. Our study also demonstrated X chromosome heterochromatin divergence among mosquito species with post-zygotic isolation, which manifests in species-specific localization of repetitive DNA as well as size and number of heterochromatin bands. The identified differences in heterochromatin support the basal phylogenetic position of *An. merus* and point to the role of heterochromatin in speciation. The differences in molecular organization of the sex chromosome heterochromatin may impair meiotic synapsis during meiosis in sterile inter-species male hybrids. We suggest that inter-species divergence of heterochromatin structure represents a cytogenetic threshold that triggers the evolutionary shift from prezygotic to postzygotic isolation. The new idiograms developed here for the sibling species will aid in future studies of heterochromatin organization and evolution in these malaria mosquitoes.

## Supplementary Materials

The following are available online at https://www.mdpi.com/2073-4425/11/3/327/s1, Figure S1: FISH of 18S rDNA with the X chromosomes in strains of *An. gambiae* and *An. coluzzii*. (**A**) *An. gambiae* strains: PIMPERENA, ZANU, KISUMU. (**B**) *An. coluzzii* strains: MALI, SUA, MOPTI. Scale bar = 1 µM. Red = 18S rDNA, Blue = DAPI., Figure S2: Fluorescence intensities of X chromosome heterochromatin in three strains of *An. gambiae* and three strains of *An. coluzzii*. (**A**) Fluorescence intensities of the proximal heterochromatin band. (**B**) Fluorescence intensities of the distal heterochromatin band., Table S1: Mosquito strains used in the study of the X chromosome heterochromatin., Table S2: Probes and primer sequences used for FISH., Table S3: Length of mitotic chromosomes in species of the *An. gambiae* complex. Table S4: Statistical analyses of the X chromosome lengths using a nonparametric Kruskal-Wallis rank sum test followed by a Dunn’s test., Table S5: Statistical analyses of the chromosome lengths using Tukey’s honestly significant difference test.
