# Supplementary material for: Structural Variation of the X Chromosome Heterochromatin in the Anopheles gambiae Complex
Source: Genes (Basel). 2020 Mar 19;11(3):327. doi: 10.3390/genes11030327 (PMC7140835; doi:10.3390/genes11030327)
Supplement: Supplementary file 1 [file genes-11-00327-s001.pdf]

Supplementary File 1

This file contains

- Supplementary Table S1-S5

- Supplementary Figures S1-S2

- Supplementary References

Table S1. Mosquito strains used in the study of the X chromosome heterochromatin.

Strain	Species	BEI ID	Isolation place	Isolation date
DONGOLA	*Anopheles arabiensis*	MRA-1235	Dongola, Sudan	2009
MALI	*Anopheles coluzzii*	MRA-860	Niono, Mali	2005
MOPTI	*Anopheles coluzzii*	MRA-763	N'Gabacoro Droit, near Bamako, Mali	2003
SUA	*Anopheles coluzzii*	MRA-765	Suakoko, Liberia	1987
KISUMU	*Anopheles gambiae*	MRA-762	Kisumu, Kenya	1975
PIMPERENA	*Anopheles gambiae*	MRA-861	PIMPERENA region, Mali	2005
ZANU	*Anopheles gambiae*	MRA-594	Zanzibar, Tanzania	1982
MAF	*Anopheles merus*	MRA-1156	Kruger National Park, South Africa	1991
SANGWE	*Anopheles quadriannulatus*	MRA-1155	Sangwe, Zimbabwe	1998

Table S2. Probes and primer sequences used for FISH.

Sequence name	GenBank accession number	PCR primer sequences	Reference to primer design

**18S rDNA**	AM157179	F: AACTGTGGAAAAGCCAGAGC<br>R: TCCACTTGATCCTTGCAAAA	[1]
**AgY53A**	AY754117	F: ATGAAGAATATGGATAATGGAT<br>R: ACGGGAGAGAGCAAGAACA	[2]
**AgY477-AgY53B**	AY754156	F: CCTTTAAACACATGCTCAAATT<br>R: GTTTCTTCATCCTTAAAGCCTAG	[2]
**Ag53C**	AY754194	F: GAACCTCTGGGCAATTTT<br>R: TCAGGATGACCATCGAAC	[2]
**AgY477**	KP666114	F: TTTGAGCATGTGTTTAAAGG<br>R: AGGTTTTCCCGAGTACAAT	[2]

Table S3. **Length of mitotic chromosomes in species of the *An. gambiae* complex.**

Species	Chromosome length, μm		
	X	2	3
	2.86	5.75	4.87
	2.69	5.85	4.26
	2.86	5.59	4.45
	2.84	5.89	4.55
	1.93	5.36	4.12
	1.91	5.23	4.08
	1.85	5.36	3.99
	1.85	5.23	3.99
	1.85	4.77	3.33
	1.79	4.7	3.39
	2.04	5.05	4
An. coluzzii MOPTI	2.14	5.1	4
	1.7	3.38	3.3
	1.89	3.91	3.09
	1.83	4.08	3.3
	1.68	4.05	3.09
	1.92	5.33	3.54
	1.84	4.86	3.49
	1.89	5.33	3.55
	1.89	4.86	3.55
	1.83	5.96	4.32
	1.9	5.98	4.26
	2.04	5.3	4.32

	1.98	5.45	4.26
	2.49	4.89	4.18
	2.6	4.77	3.78
	1.98	4.89	4.18
	2.16	5.1	3.78
	1.89	4.43	3.19
	2	4.31	3.31
	2.11	4.99	3.68
	1.95	5	3.69
	1.89	5.29	4.59
	1.85	5.78	4.59
	1.91	5.43	4.37
	1.92	5.52	3.99
	1.75	5.06	4.06
	1.98	5.09	4.08
	1.66	5.06	4.06
	1.62	5.09	4.08
An. arabiensis	3.98	15.74	12.71
	2.69	6.1	4.7
	2.6	5.76	4.91
	2.04	3.46	3.81
	2.02	3.33	3.51
	1.85	3.55	3.18
	1.8	3.33	2.86
	3.79	4.91	4.58
	3.13	4.69	4.35
	2.06	3.67	3.28
	2.16	3.62	3.28
	2.24	4.43	3.68
	2.18	4.38	3.58
An. quadriannulatus	2.56	4.52	3.79
	2.88	4.2	3.8
	2.3	4.68	3.72
	2.35	4.91	4.33
	2.38	4.67	3.91
	2.27	4.65	3.92
	3.15	6.05	5.12
	3.62	5.61	5.11
	4.25	6.08	5.72
	2.04	3.68	3.61
	2.27	3.82	3.77
An. merus	4.97	6.15	4.75
	5.51	6.04	5.86

5.57	8.69	6.13
5.6	5.22	4.71
5.34	4.97	3.82
5.16	4.82	3.93
5.33	5.85	5.23
6.02	6.06	5.12
5.64	5.68	4.37
5.76	6.21	4.44
4.79	4.44	3.53
4.94	5.03	4.14

Table S4. Statistical analyses of the X chromosome lengths using a nonparametric Kruskal-Wallis rank sum test followed by a Dunn's test.

Levene’s Test for Homogeneity of Variance: p-value: 0.05465

Kruskal-Wallis chi-squared = 44.624, df = 3, p-value = 1.112e-09

Dunn’s test:

```
Col Mean-|
Row Mean |   An. arab   An. coluz   An. meru
---------+---------------------------------
An. colu |   2.379984
         |   0.0087*
         |
An. meru |  -3.294863  -6.315910
         |   0.0005*    0.0000*
         |
An. quad |  -1.028104  -3.468913   2.150846
         |    0.1520    0.0003*    0.0157*
alpha = 0.05
Reject Ho if p <= alpha/2
```

Table S5. Statistical analyses of the chromosome lengths using Tukey's honestly significant difference test.

Chromosome X (TukeyHSD):

	diff	lwr	upr	p adj
An. coluzzii-An. arabiensis	-0.4840769	-0.8853592	-0.08279465	0.0116598
An. merus-An. arabiensis	2.8827564	2.3795787	3.38593414	0.0000000
An. quadriannulatus-An. arabiensis	0.2305594	-0.2843743	0.74549314	0.6428440
An. merus-An. coluzzii	3.3668333	2.9531245	3.78054214	0.0000000
An. quadriannulatus-An. coluzzii	0.7146364	0.2867066	1.14256609	0.0002180
An. quadriannulatus-An. merus	-2.6521970	-3.1768723	-2.12752162	0.0000000

Chromosome 2 (TukeyHSD):

	diff	lwr	upr	p adj
An. coluzzii-An. arabiensis	-0.07478846	-1.3416783	1.1921014	0.9986545
An. merus-An. arabiensis	0.61179487	-0.9767895	2.2003793	0.7424397
An. quadriannulatus-An. arabiensis	-0.34517483	-1.9708740	1.2805243	0.9439505
An. merus-An. coluzzii	0.68658333	-0.6195383	1.9927050	0.5143073
An. quadriannulatus-An. coluzzii	-0.27038636	-1.6214050	1.0806322	0.9524360
An. quadriannulatus-An. merus	-0.95696970	-2.6134243	0.6994849	0.4313540

Chromosome 3 (TukeyHSD):

	diff	lwr	upr	p adj
An. coluzzii-An. arabiensis	-0.5768654	-1.5585237	0.4047929	0.4161296
An. merus-An. arabiensis	0.1745513	-1.0563742	1.4054768	0.9821631
An. quadriannulatus-An. arabiensis	-0.2400699	-1.4997541	1.0196142	0.9585231
An. merus-An. coluzzii	0.7514167	-0.2606407	1.7634740	0.2156810
An. quadriannulatus-An. coluzzii	0.3367955	-0.7100506	1.3836415	0.8321909
An. quadriannulatus-An. merus	-0.4146212	-1.6981364	0.8688940	0.8304914

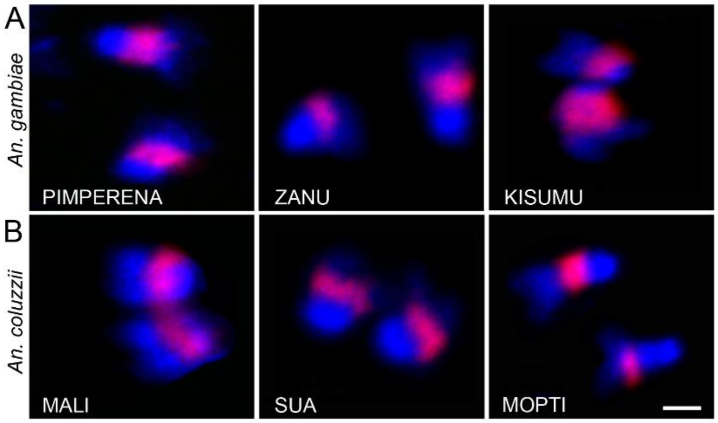

Figure S1. FISH of 18S rDNA with the X chromosomes in strains of *An. gambiae* and *An. coluzzii*. (**A**) *An. gambiae* strains: PIMPERENA, ZANU, KISUMU. (**B**) *An. coluzzii strains:* MALI, SUA, MOPTI. Scale bar – 1 μM. Red – 18S rDNA, Blue – DAPI.

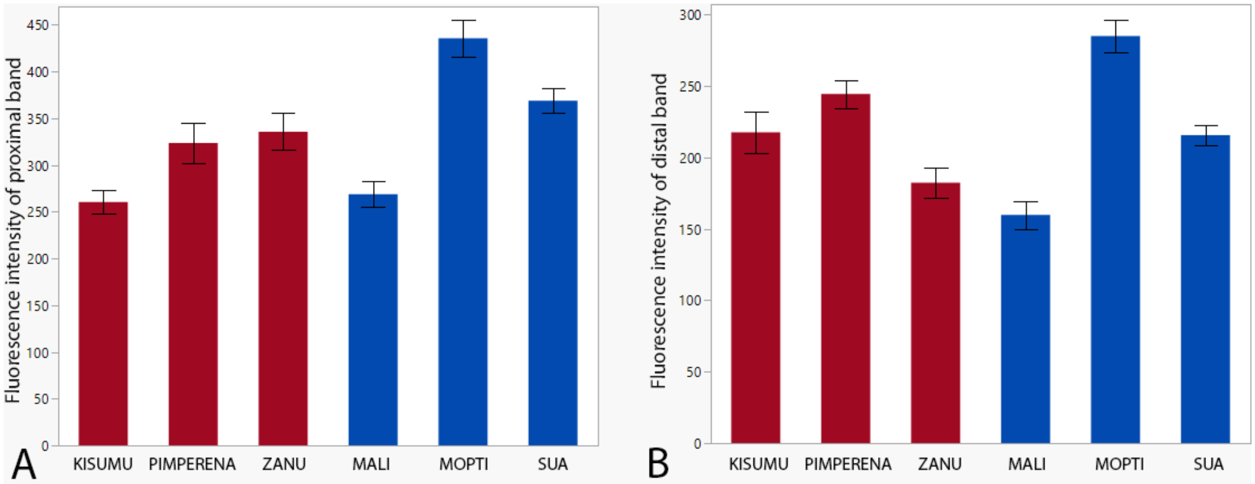

Figure S2. Fluorescence intensities of X chromosome heterochromatin in three strains of *An. gambiae* and three strains of *An. coluzzii*. (**A**) Fluorescence intensities of the proximal heterochromatin band. (**B**) Fluorescence intensities of the distal heterochromatin band.

Supplementary References

1. Liang, J.; Sharakhov, I.V. Premeiotic and meiotic failures lead to hybrid male sterility in the Anopheles gambiae complex. *Proc Biol Sci* **2019**, *286*, 20191080, doi:10.1098/rspb.2019.1080.
2. Krzywinski, J.; Sangare, D.; Besansky, N.J. Satellite DNA from the Y chromosome of the malaria vector Anopheles gambiae. *Genetics* **2005**, *169*, 185-196.
